# A Concise History of the Principal Discoveries in Anatomy

**Published:** 1800-04

**Authors:** 


### A Concise History of the Principal Discoveries in Anatomy.

[ Continued from our last Number, pp. 256—261. ]

VAROLI, next to MASSA, described the first pair better than his predecessors, whom he justly reprobated for having entertained so imperfect an idea of the olfactory nerves. He traced their origin to the sulcus of the anterior lobes of the brain, stated their use to consist merely in conveying the sensation of odours, without contributing to the secretion of the humours from the ventricles of the brain; but he furnished us with no faithful drawing of this nerve.* Next to him PICCOLHUOMINI represented this first pair with tolerable exactness.†

§. 36. With regard to the optic nerves, we shall observe that EUSTACHIUS was the first, after GALEN, who could claim the merit of having explained in the best manner, by engravings, the origin of these nerves from their *thalami*, on each side of the septum, and between the *crura medullæ oblongatæ*.‡ The credit therefore is not due to VAROLI, who ascribes to himself the discovery of the thalami nervorum opticorum in the year 1570; and the account of his controversy with other anatomists, who were unable to trace, according to his directions, the origin of these nerves, is no less singular.§ FABRICIUS likewise accurately describes their origin from the vicinity of the corpora quadrigemina, and between the crura medullæ oblongatæ.‖ The decussation of the optic nerves, which had been denied by GALEN, also occasioned a very careful investigation in the sixteenth century. VESALIUS particularly informs us of observations having been made, which prove that, after the loss of sight on the right eye, the nerve of that side had been found weak and corrugated, not only as far as its union with the nerve on the opposite side, but also behind its connexion to the thalami of the right side.

VESALIUS therefore, as well as most other anatomists of this century, adopted the opinion of no decussation, but only an approximation of the nerves, or an entire union of their medullary substance, without impeding their course and disposition; consequently the nerve originating from the right side of the thalami, was said to proceed to the right, and that coming

---

* *Varol.* de nerv. optic. f. 9. a.—Anatom. lib. i. c. 5. p. 23.

† Anatom. praelect. p. 263.

‡ Tab. xvii. fig. 4. *(M M.)* particularly fig. 6. *(O P.)*

§ De nerv. opt. f. 13. a. b. ‖ De oculo, p. 193.

ing from the left, to extend to the left eye.* A complete union of the medullary substance, without decussation, was admitted by STEPHANUS,† COLUMBUS,‡ BAUHINUS,§ and VAROLIUS;‖ but a mere approximation only, by FABRICIUS.¶

With respect to the structure of the optic nerve, the ancient authors erroneously believed it to be tubular, and asserted that the use of this cavity consisted in conducting the *spiritus visorius* to the eye. This error was probably occasioned by a similar observation on the central artery; it was rectified in the earlier part of the sixteenth century. We are told by BERENGAR, that he has bestowed great pains in detecting the canal in the optic nerve, but almost constantly without success. Once indeed he observed a cavity in the optic nerve of a swine; when he says, "*ipsi nervi nempe erant concavi, sicut vena seu arteria*:" it likewise appeared to him as if a vacuum existed in the interior part where the optic nerves are connected; but in the optic nerves of man not the smallest cavity could be found, either on one or the other side of their union. The porosities in these nerves, he concludes, must therefore, in all probability, not be much larger than those of others, as the *spiritus visorius* is so very subtle and penetrating. The substance of the optic nerves is, according to him, soft and medullary** VESALIUS, who examined the optic nerve not only in different animals, but also in a man immediately after his decapitation, was equally unsuccessful in discovering any cavity, not even in the place of their union.†† PUTEUS, however, asserted that these pori were visible in the optic nerves of cattle;‡‡ yet VESALIUS maintained that the optic nerve was merely of a fibrous structure, and ironically ascribed it to his want of attention, that he had hitherto been unable to discover those pretended pores.§§ In this he also was seconded by FALLOPIUS,‖‖ and COLUMBUS.¶¶ Yet both these authors asserted that the optic nerves were of a porous, or rather loose structure, in order to admit the free access of the *spiritus visorius*; and DU LAURENS even affirmed that they consisted of a spongy substance.*** COITERUS assures us, that the optic nerve was composed of mere fibres, and consequently could not

* *Vesal.* lib. iv. c. 4. p. 366.

† *Stephan.* p. 293.

‡ Lib. viii. c. 3. p. 358.

§ Theatr. p. 648.

‖ Anatom. lib. i. c. 4. p. 14.

¶ De oculo, p. 239.

** *Berengar.* f. 452. b.

†† Lib. iv. c. 4. p. 366.

‡‡ Apolog. f. 92. a.

§§ De radic. chyn. p. 660.

‖‖ Obs. p. 402.

¶¶ Lib. viii. c. 3. p. 358.

*** *Laurent.* lib. iv. c. 16. p. 276.

not be of a tubular nature.* Neverthelefs, the old hypothefis was maintained by three of the moft celebrated authors of the fixteenth century, who, as has already been obferved, were mifled by the appearance of the central artery, to admit a hollow form of the optic nerve. EUSTACHIUS declares he has, at innumerable times, fubmitted the cavity of the optic nerve to the infpection of fceptics, and thereby filenced their doubts.† ARANZI maintains that, in the eyes of fubjects recently deceafed, he could, without the leaft difficulty, introduce a needle into the cavity;‡ and GUIDI § fays, that he has clearly obferved a cavity at the iffue of the optic nerve into the retina, though it could not be farther traced in its progrefs. Finally, FABRICIUS doubts the exiftence of fuch a hole, but will not give a decifive opinion upon the fubject.||

§. 37. The origin of the third pair of nerves was accurately pointed out by VAROLIUS ¶, who traced it from the medullary clufters of the cerebrum, with extremely clofe and often united fibrils. VESALIUS improperly delineates its courfe and extenfion, when he pretends that its ramifications proceed to all the mufcles of the eye.** COLUMBUS corrected this error by allowing two mufcles, the rectus abducens, and the obliquus fuperior; but, at the fame time, he committed another, when he believed that this nerve extended alfo to the temporal mufcle, and that the fympathy fubfifting between the eyes and the temples, may thence be explained.†† FALLOPIUS very jufty cenfured COLUMBUS for this miftake;‡‡ who, in diffecting the *ganglion opthalmicum*, was mifled to follow its progrefs from the branches of the third pair, which affift in forming this ganglion, through the lachrymal nerve to the profound nerve of the temporal mufcle (from the third branch of the fifth pair), and thus to confider the latter as a continuation of the third pair. FALLOPIUS, at the fame time, corrected the error of VESALIUS, and fhewed that thofe two above mentioned mufcles of the eye were not influenced by this nerve. But VESALIUS did not manifeft, in his anfwer, that degree of confidence which the accurate demonftrations of FALLOPIUS amply deferved.§§

* Tab. oculor. p. 87.

† Off. exam. p. 205.

§ *Vid.* lib. iii. c. 1. p. 80.

|| De oculo. p. 238.

¶ De nerv. optic. f. 13. b.—Compare *Laurent.* hift. anat. lib. xi. c. 8. p. 928.

** Lib. iv. c. 5. p. 367.

†† Lib. viii. c. 3. p. 359.

‡‡ Obf. p. 402.

§§ Exam. obf. Faliop. p. 803.

§ 38. The fourth pair, or the pathetic nerves, were apparently known to ACHILLINI, when he traced from the posterior part of the brain a new nerve, never taken notice of before his time; described it as very thin; and believed that it terminated in the eyebrows.* He was probably misled to form this last erroneous conjecture, by having observed that the pathetic nerve frequently unites with the first and principal branch of the fifth pair. It does not at first appear, whether VESALIUS was acquainted with this nerve; he assigns to his third (or what is at present considered as the fifth pair) a double root, one very tender, and the other very solid.† The tender root is, according to him, so distributed, that we may be induced to conclude it to be the first chief branch of the fifth pair;‡ but the principal objection is the pretended origin of this tender root, which is said to arise from the posterior part of the brain, where it extends to the spinal marrow; this nerve, it is farther maintained, does not combine with what is strictly called the third (or the present fifth pair), and we may therefore justly consider it as a separate nerve. He would not, however, venture so far as positively to defend this last proposition, on account of the established order. The same assertion cannot be applied to the first and principal branch of the fifth pair. The account given by FALLOPIUS, relative to the pathetic nerve, is worthy of observation, viz. that it was described by VESALIUS under the name of the tender root of the third pair; but he attributed to it too many ramifications,§ and that VESALIUS in his reply acknowledged himself to have stated this ramification beyond its natural course.‖ Hence we may infer, that VESALIUS has accurately seen the origin of the nerve, and traced it to a connection with the first of the principal branches belonging to the fifth pair, but afterwards confounded it with the latter. FALLOPIUS was the first, who with due precision called this nerve the *eighth*, and stated its origin to be behind the posterior corpora quadrigemina, and its distribution only to the obliquus superior of the eye. EUSTACHIUS has likewise given a representation of it,¶ but has mentioned it rather obscurely.** COLUMBUS denominates the same nerve the *ninth*, and undeservedly assumes the merit of its discovery.†† GUIDI has adopted the description given by FALLOPIUS.‡‡

§. 39.

---

* *Achillini* annot. in Mundin. p. 13. † *Vesal.* lib. iv. c. 6. p. 367.

‡ *Meckel.* de quinto pare. s. 5.

§ *Fallop.* obs. p. 403.—Compare *Morgagni* ep. anat. xv. s. 45.—*Sömmerring* de basi encephali. s. 51. ‖ *Vesal.* exam. obs. Fallop. p. 803.

¶ *Eustach.* tab. xvii. fig. 2. (*MMN.*)

** Oss. exam. p. 205. "Nervus, qui prope nares exoritur."

†† Lib. viii. c. 3. p. 365. ‡‡ *Vid.* lib. iii. c. 1. p. 83.

§ 39. The history of the *fifth pair*, evinces, in the most striking manner, that neurology, the most difficult part of anatomy, has, by very slow degrees, and many deviations from the true path, arrived at its present state of perfection. In the description of this pair, as attempted by BERENGAR, we meet with much confusion: he divides it into two single nerves, which, according to the example of the old school, constitute the third and fourth pair. From his third pair, the first branch descends near the *carotis*, along the vertebræ of the neck, through the diaphragm, into the abdominal viscera. BERENGAR has probably, in this instance, pursued the ramus profundus of the nervus Vidianus, which combines with the intercostal nerve, as well as with the last mentioned nerve itself. The other branches of his third pair proceed to the eyes, the nose, the temporal muscles, those of the face, and combine with his fifth, or the facial, nerve. The fourth nerve of BERENGAR is evidently our common trunk of the Vidian and palatine nerves.* The description of VESALIUS is rendered the more intricate, as he at the same time examines what is now called the fourth pair, while he considers the common trunk of the Vidian and palatine nerves as a particular nerve, by the name of the fourth. He divides his third pair of nerves into a soft and a firm portion; the former proceeding with four branches to the forehead, the upper jaw, the muscles of the lips, and to the temples. VESALIUS has probably not paid sufficient attention in dissecting this branch, but traced its ramus lachrymalis to the temples, which, however, are supplied by the second branch of the fifth pair. The second and third principal branches he calls the thick portion of the third pair, but separates from it, as has been before observed, the common trunk of the Vidian and palatine nerves. The distribution of the thick portion is correctly stated, except the infraorbital nerve, which has been omitted. The nervus lingualis, which he derives from the firm portion, is, in his opinion, the peculiar nerve for regulating the sense of taste. † MASSA describes the fifth pair by the name of the fourth, fifth and sixth.‡ But the description of FALLOPIUS is more correct. He divides our fifth, or his third pair, into three branches, and the first main branch into two, either entirely excluding the lachrymal branch, or deriving it from the *naso-ocularis*. The latter, according to him, unites in its ramifications with the optic nerve; though his opinion be not altogether conformable to truth. He is

* *Berengar*. commentar. in Mundin. f. 456. b. 457. a.

† *Vesal*. lib. iv. c. 6. p. 367. ‡ Introduct. p. 79.

is well acquainted with the maxillaris superior, and its passage through the maxillary bone. He supposes detached branches to proceed from the buccinatorius to the fauces. This mistake arose probably from the circumstance that the buccinator muscle is connected with the cephalo-pharyngeus. He represented very correctly the cord which the temporal nerve form round the arteria meningea, as well as the nervus *temporalis superficialis.** COLUMBUS adheres to the division adopted by FALLOPIUS;† yet he separates, first, the *massetericus*, (like PALETTA,‡) from the fifth pair of ours, and calls it the eighth. GUIDI has more distinctly described the common trunk of the Vidian and palatine nerves, in commemoration of which the pterygoideus has been called nervus Vidianus.§

§ 40. The *sixth pair of nerves*, which is so important by its combination with the intercostal nerve, was, to our knowledge, first discovered by EUSTACHIUS, who gave a precise account of its origin, progress and union with the intercostal nerve.‖ The more minute division which VESALIUS makes of the fifth pair, cannot (in the opinion of Professor Sprengel) be considered as a sixth pair; but, guided by EUSTACHIUS, several anatomists have correctly pointed out its combination with the intercostal nerve. FALLOPIUS, however, has, without mentioning this combination, accurately described its ramification to the abductor oculi.¶

As the *acoustic nerve* not only combines through a loose cellular membrane with the *facial nerve*, a common canal in the os temporis from the cranium, proceeds along with it, forms the chorda tympani, and likewise supplies several muscles of the auditory organ; it is a very pardonable error that the two nerves were at those times considered as branches of one common trunk,** which went by the appellation of the *fifth* pair. On this occasion, the distribution of the acoustic nerve was usually not attended to, while, on the other hand, the facial nerve was the more fully described. VESALIUS gives a brief,

* *Fallop.* obs. p. 403, 404.

† Lib. viii. p. 365.—It would appear as if Columbus described what is now called the sixth pair of nerves, under the name of the eighth. This is likewise asserted by Pfeffinger, in his work "De Structur. Nerv. sect. 2. § 21." But on a more accurate investigation, we shall find that his description may, with more propriety, be applied to the *massetericus*.

‡ *Paletta* de nervis crotaphit. et buccinator. in *Romer.* delect. opuscul. vol. i. p. 113. f.

§ *Vid.* lib. iii. p. 81.

‖ Tab. xviii. fig. 1. 3. 5. *(o.)* particularly fig. 2. *(Z Z. ξξ.)*

¶ *Fallop.* obs. p. 405.

** *Berengar.* f. 457. b.—*Vesal.* lib. iv. c. 8. p. 368.

but tolerably correct account of its union with the second branch of the fifth pair, its distribution to the muscles of the auditory organ, and its subsequent grand ramification to all the muscles of the face. EUSTACHIUS, indeed, still believes that the facial nerve is a branch of the acoustic nerve, though he likewise is at the same time, acquainted with the three portions of the latter, and has observed the connection of the chorda tympani, which proceeds from the facial nerve, with the nervus lingualis (from the third branch of the fifth pair).* In this instance also, FALLOPIUS was a more accurate observer than all his cotemporaries. He was convinced that the facial nerve formed a peculiar pair; but that he might not appear singular, he maintained the old division.† Although VAROLI discovered the origin of the acoustic nerve in the medullary ganglion,‡ *(pons Varolii)*, and PICOLHUOMINI found the roots of the fifth pair in the fourth ventricle of the brain,§ yet the latter discovery rather applies to what we at present call the acoustic nerve, and the former may be referred to the facial nerve. From the union of the lingual nerve with the chorda tympani, VAROLI explained the phenomenon that deaf persons are commonly also subject to the loss of speech.||

[ To be concluded in our next Number. ]

* *Eustach.* de audit. organ. p. 136, 141.—Tab. xviii. fig. 1. *(R T Z.)* and fig. 3. *(T.)*—Compare *Coiter* p. 99.—*Ingrass.* comm. in *Gal.* de oss. p. 9.

† Obs. p. 405.—Compare *Coiter*, p. 104.

‡ De nerv. optic. f. 4. a.

§ Anatom. praelect. p. 300.

|| Anatom. lib. i. c. 7. p. 31.

